# NCT/DKFZ MASTER handbook of interpreting whole-genome, transcriptome, and methylome data for precision oncology

**DOI:** 10.1038/s41698-023-00458-w

**Published:** 2023-10-26

**Authors:** Andreas Mock, Maria-Veronica Teleanu, Simon Kreutzfeldt, Christoph E. Heilig, Jennifer Hüllein, Lino Möhrmann, Arne Jahn, Dorothea Hanf, Irina A. Kerle, Hans Martin Singh, Barbara Hutter, Sebastian Uhrig, Martina Fröhlich, Olaf Neumann, Andreas Hartig, Sascha Brückmann, Steffen Hirsch, Kerstin Grund, Nicola Dikow, Daniel B. Lipka, Marcus Renner, Irfan Ahmed Bhatti, Leonidas Apostolidis, Richard F. Schlenk, Christian P. Schaaf, Albrecht Stenzinger, Evelin Schröck, Daniel Hübschmann, Christoph Heining, Peter Horak, Hanno Glimm, Stefan Fröhling

**Affiliations:** 1 Division of Translational Medical Oncology, National Center for Tumor Diseases (NCT) Heidelberg and German Cancer Research Center (DKFZ), Heidelberg, Germany; 2 Department of Hematology, Oncology and Rheumatology, Heidelberg Unversity Hospital, Heidelberg, Germany; 3 Computational Oncology Group, Molecular Precision Oncology Program, NCT Heidelberg and DKFZ, Heidelberg, Germany; 4 Faculty of Medicine and University Hospital Carl Gustav Carus, Technische Universität Dresden, Dresden, Germany; Helmholtz-Zentrum Dresden-Rossendorf (HZDR), Dresden, Germany; 5 Translational Medical Oncology, Faculty of Medicine and University Hospital Carl Gustav Carus, Technische Universität Dresden, Dresden, Germany; 6 Department of Translational Medical Oncology, National Center for Tumor Diseases/University Cancer Center (NCT/UCC) Dresden, Dresden, Germany; 7 DKFZ, Heidelberg, Germany; 8 Institute for Clinical Genetics, University Hospital Carl Gustav Carus, Technische Universität Dresden and Hereditary Cancer Syndrome Center Dresden, Dresden, Germany; 9 Department of Medical Oncology, NCT Heidelberg and Heidelberg University Hospital, Heidelberg, Germany; 10 Institute of Pathology, Heidelberg University Hospital, Heidelberg, Germany; 11 Institute of Pathology, Faculty of Medicine and University Hospital Carl Gustav Carus, Technische Universität Dresden, Dresden, Germany; 12 Institute of Human Genetics, Heidelberg University Hospital, Heidelberg, Germany; 13 Translational Cancer Epigenomics, Division of Translational Medical Oncology, NCT Heidelberg and DKFZ, Heidelberg, Germany; 14 NCT Trial Center, NCT Heidelberg and DKFZ, Heidelberg, Germany; 15 Present Address: Institute of Pathology, Ludwig-Maximilians-Universität (LMU) München, Munich, Germany

**Keywords:** Cancer genomics, Translational research

## Abstract

Analysis of selected cancer genes has become an important tool in precision oncology but cannot fully capture the molecular features and, most importantly, vulnerabilities of individual tumors. Observational and interventional studies have shown that decision-making based on comprehensive molecular characterization adds significant clinical value. However, the complexity and heterogeneity of the resulting data are major challenges for disciplines involved in interpretation and recommendations for individualized care, and limited information exists on how to approach multilayered tumor profiles in clinical routine. We report our experience with the practical use of data from whole-genome or exome and RNA sequencing and DNA methylation profiling within the MASTER (Molecularly Aided Stratification for Tumor Eradication Research) program of the National Center for Tumor Diseases (NCT) Heidelberg and Dresden and the German Cancer Research Center (DKFZ). We cover all relevant steps of an end-to-end precision oncology workflow, from sample collection, molecular analysis, and variant prioritization to assigning treatment recommendations and discussion in the molecular tumor board. To provide insight into our approach to multidimensional tumor profiles and guidance on interpreting their biological impact and diagnostic and therapeutic implications, we present case studies from the NCT/DKFZ molecular tumor board that illustrate our daily practice. This manual is intended to be useful for physicians, biologists, and bioinformaticians involved in the clinical interpretation of genome-wide molecular information.

## Introduction

Precision oncology (PO) is an emerging, highly interdisciplinary field of cancer medicine that aims to develop and apply clinical management strategies tailored to individual patients’ biological characteristics^1,2^. It has grown rapidly with the widespread availability of next-generation sequencing-based methods for detecting acquired molecular alterations that drive tumor growth^3^. In parallel, the need to identify hereditary factors that predispose to cancer development has also increased^4^. Structurally, the importance of PO is reflected in the growing number of cancer centers maintaining dedicated molecular tumor boards (MTBs) for biologically guided clinical decision-making^5–10^. Most PO workflows have been built around the analysis and interpretation of subgenomic cancer gene panels^11^, and a number of position papers offer guidance in interpreting the biological effects and clinical implications of cancer variants^12–15^. This handbook aims to support the advancement of PO by (i) describing the experience gained in the clinical interpretation of data from multidimensional tumor characterization by whole-genome or exome (WGS/WES) and RNA sequencing (RNA-seq) and DNA methylation profiling in the MASTER (Molecularly Aided Stratification for Tumor Eradication Research) trial of the National Center for Tumor Diseases (NCT) and the German Cancer Research Center (DKFZ)^6,16^ and (ii) presenting key concepts using clinical cases from the MTB at NCT Heidelberg/Dresden.

## Results

### Patient characteristics and tissue context

**Fig. 1:**
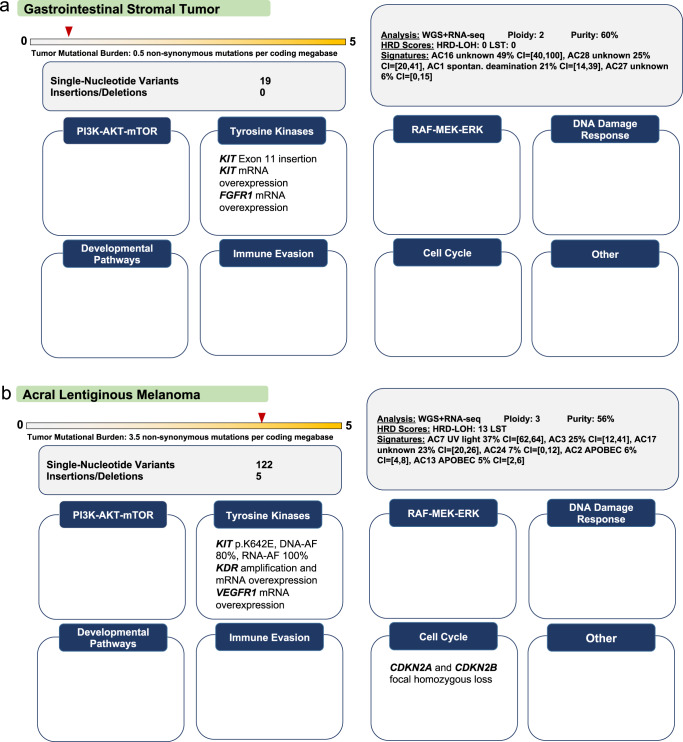
Impact of patient characteristics and tissue context on the clinical interpretation of molecular alterations. Cases were selected to exemplify how to approach, following current guidelines for oncogenicity classification, somatic variants that have not been curated and to emphasize the therapeutic impact of the histologic context. In addition, we included a structural variant, i.e., an insertion, to illustrate curation challenges in daily routine. **a** Gastrointestinal stromal tumor (GIST) studied by WES and RNA-seq of formalin-fixed and paraffin-embedded tumor tissue (histopathologic tumor cell content, 50%). A *KIT* exon 11 insertion (p.P585_R586insSPYDHKWEFP), whose expression was verified by RNA-seq, was nominated as a candidate driver because in-frame indels in *KIT* exon 11, encoding the KIT juxtamembrane domain, are known oncogenic events in GIST and rarely occur in other cancers (www.cancerhotspots.org^55^). A literature search revealed that a similar variant (p.P585_586insLPYDHKWEFP) was detected in a previous study but has not been functionally characterized to date^63^. Application of the VICC guideline for interpreting somatic variants in tumors (www.cancervariants.org^28^) resulted in a score of seven points, classifying the variant as likely oncogenic, which was composed of evidence from the following categories: “Oncogenicity Moderate 1” (OM1; two points): variant located in a critical and well-established part of a functional domain; OM2 (two points): variant associated with protein length changes because of in-frame indels in a known oncogene or tumor suppressor gene or stop-loss variants in a known tumor suppressor gene; “Oncogenicity Supporting 3” (OP3; one point): variant absent from controls or occurring at an extremely low frequency in the Genome Aggregation Database (gnomAD; https://gnomad.broadinstitute.org); OP4 (one point): variant located in a mutation hotspot listed in Cancer Hotspots (www.cancerhotspots.org) and associated with an amino acid change count in Cancer Hotspots below 10 (resources such as cBioPortal [www.cbioportal.org], COSMIC [https://cancer.sanger.ac.uk/cosmic], or an entity’s published genetic landscape to be used for variants occurring in tumor types not covered well by Cancer Hotspots). Furthermore, we added OP2 evidence (one point; variant in a gene in a malignancy with a single genetic etiology) because *KIT* mutations drive the vast majority of GIST, and exon 11 indels are among the recurrent alterations. Based on this evaluation, the MTB recommended therapy with imatinib with an NCT evidence level of m1a, because *KIT* exon 11-mutant GIST is particularly sensitive to this agent^64^. CI, confidence interval; SBS, single-base substitution. **b** RAF- and NRAS-wildtype acral melanoma studied by WGS and RNA-seq of fresh-frozen tissue (histopathologic tumor cell content, 65%) after progressing on immuncheckpoint inhibition. The *KIT* gene was affected by a p.K642E missense mutation with loss of heterozygosity and an allele frequency (AF) of 80%, whose expression was verified by RNA-seq (AF, 100%), and a DNA copy number of 8 (average ploidy, 3). Activating *KIT* mutations occur in approximately 3% of melanomas, with enrichment in the acral subtype, and include mainly missense mutations affecting exons 9, 11, 13, 17, and 18, with up to 60% occurring in exons 11 or 13. The p.K642E and p.L576 variants account for approximately one-quarter of *KIT* mutations in melanoma and provide a rationale for therapy with imatinib. However, the objective response and disease control rates of these patients (24.4% and 66.7%, respectively;^65^) are lower than those of patients with *KIT*-mutant GIST (80% and >90%, respectively), context-specific differences whose basis remains to be elucidated. APOBEC, apolipoprotein B mRNA editing enzyme, catalytic polypeptide; UV, ultraviolet. Take-home messages: (i) Current VICC guidelines should be applied when evaluating somatic variants of unclear biological significance. Certain alteration types remain difficult to annotate and may require case-by-case assessment, which should take place in multidisciplinary MTBs whenever possible. (ii) The clinical actionability of a driver alteration, determined by, e.g., the probability and duration of response to molecularly guided therapy, can vary widely depending on the histologic context, which must always be considered when selecting and prioritizing treatment options.

The clinical interpretation of molecular alterations starts with evaluating relevant patient characteristics and the tissue context in which a genetic profile occurs. The former relates, in particular, to previous therapies, in addition to disease stage and clinical performance status. For example, prior targeted therapies warrant a search for possible resistance mutations, and progression on single-agent immune checkpoint inhibition requires consideration of combination therapies if the tumor exhibits predictive biomarkers for immunotherapy. Concomitant cancers and non-oncologic diagnoses are other important host factors to account for. Tissue context refers to the histologic entity, biopsy site, type of tissue preservation, i.e., formalin fixation and paraffin embedding vs. snap freezing, and preanalytical parameters such as DNA and RNA quality and tumor cell content estimated by an experienced pathologist. Each case also requires consideration of the tumor entity’s molecular landscape, e.g., recurrent mutations, copy number alterations, and gene fusions (Fig. 1).

### Quality measures, summary statistics, and complex molecular profiles

**Fig. 2:**
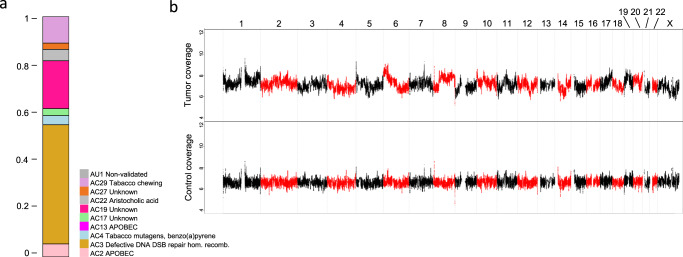
Complex biomarkers derived from WES of a peritoneal metastasis in a patient with ovarian cancer. **a** Fractions of mutational signatures identified in the tumor. DSB, double-strand break. hom. recomb., homologous recombination. **b** Somatic DNA copy number profiles of the tumor and a matched normal control. The tumor exhibits segmental gains and losses of all chromosomes as well as a high HRD-LOH score and numerous LSTs (19 and 23, respectively), corresponding to a highly rearranged genome. Consistent with the genomic “scars” of HRD, the SBS3 mutational signature explained 50% of all SNVs. Of note, no germline or somatic mutations in *BRCA1/2* were detected. Chromosomes 1 to X are indicated.

**Fig. 3:**
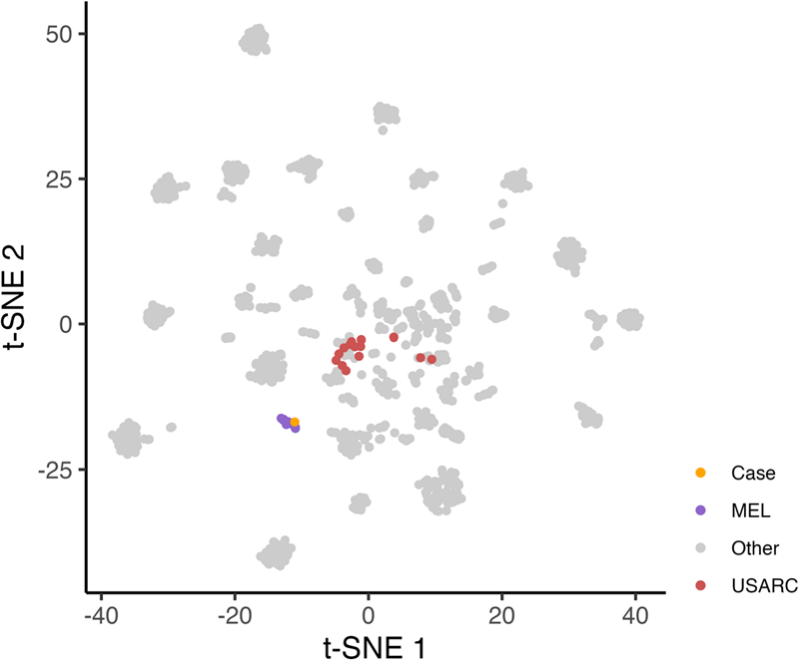
Clinical implications of DNA methylation analysis. Patient with undifferentiated pleomorphic sarcoma of the lung according to histopathology. Immunohistochemistry: Melan A, HMB45, CD34, MyoD, CD30, SOX10, CD68, CD117, cytokeratin 7/8, CD123, CD1a, and CD21 negative; CD56, S100, and PD-L1 (90%, Cologne Score 5) positive; proliferation rate (MIB-1), 80%. Treatment course: surgery followed by adjuvant chemotherapy with doxorubicin and ifosfamide; switch to pazopanib due to liver metastases, stable disease; switch to trabectedin and pembrolizumab due to lung metastases after six months, complete metabolic response; brain metastases with continuing remission at peripheral sites after six months. WGS and RNA-seq revealed gene expression similarity to melanoma, a high tumor mutational burden (891 SNVs and 8 indels), and a highly prevalent SBS7 mutational signature associated with UV light exposure^66^. DNA methylation profiling showed a match score of 0.95 with cutaneous melanoma^23,67^. The figure shows a projection of the index case and several melanomas (MEL) on a DNA methylation-based sarcoma reference cohort (*n* = 1077;^23^), in which undifferentiated sarcomas (USARC) are highlighted (t-distributed stochastic neighbor embedding [t-SNE] using the 10,000 most variable probes according to standard deviation via the R package Rtsne (version 0.16) using 3000 iterations and a perplexity value of 30). This finding prompted recommendations to reevaluate the diagnosis and modify further treatment if applicable. Take-home messages: (i) New multiomics layers, such as genome-wide DNA methylation profiles, can help refine diagnosis, especially for cancer types without distinct morphologic features or pathognomonic molecular alterations. (ii) Multiomics-guided diagnostic reclassification can inform therapeutic decision-making.

After examining general quality measures of sequencing runs, such as library size and RNA mapping and duplication rates, we first evaluate summary statistics and complex biomarkers, whose detection is enabled by comprehensive and multilayered profiling. These biomarkers include computational estimates of tumor purity and ploidy, tumor mutational burden, mutational signatures (Fig. 2a^17^), and the quantification of genomic instability by assessing the loss-of-heterozygosity-homologous-recombination-deficiency (HRD-LOH) score and the number of the large-scale state transitions (LSTs; Fig. 2b). The HRD-LOH score corresponds to the number of subchromosomal segments with loss of heterozygosity larger than 15 megabase pairs (Mbp), and LSTs are defined as switches between segments with different copy number states larger than 10 Mbp but smaller than entire chromosome arms^18–20^. Moreover, we quantify microsatellite instability according to the MSIsensor algorithm^21^. For central nervous system tumors^22^ and sarcomas^23^, genome-wide DNA methylation profiles allow entity predictions using published classifiers (Fig. 3). For all other entities, similarity analyses of transcriptional profiles within the MASTER cohort allow comparison of an individual case with known diagnoses^24^. When methylome- or transcriptome-based entity predictions suggest a likely differential diagnosis, pathologic reevaluation is recommended.

### Highly actionable and entity-defining alterations

The first individual molecular changes we evaluate from a clinical perspective are the “known knowns” of PO, i.e., highly actionable and entity-defining alterations. This is facilitated by a whitelist in our variant annotation pipeline consisting of (i) genes that are part of the OnkoKB knowledge base^25^, (ii) biomarker-drug associations that were the basis of previous MTB recommendations at NCT Heidelberg/Dresden, and a manually curated set of entity-defining alterations, e.g., *SS18::SSX* fusions in synovial sarcoma^26^. This whitelist is continuously adapted as new evidence becomes available, and clinical interpretation is not limited to this gene set. Even if convincing evidence for treatment recommendations can be provided based on the highly actionable genes alone, we seek to explore all biological layers to identify new parameters that can inform clinical management. For example, SNVs or copy number alterations are always presented alongside the respective gene’s expression level to allow for integrative interpretation. All clinically actionable alterations are assigned to seven biomarker baskets based on the cellular pathways or processes involved: tyrosine kinases, PI3K-AKT-mTOR signaling, RAF-MEK-ERK signaling, cell cycle, developmental regulation, DNA damage repair, and immune evasion.

### Oncogenicity of small genetic variants

We regularly encounter genetic variants in known cancer genes that have not been described in PO knowledge bases^27^. In such cases, we apply the VICC standard operating procedure for interpreting the pathogenicity of somatic variants in cancer^28^. It focuses on the oncogenicity of acquired small genetic alterations, i.e., SNVs and indels, but is not intended for interpreting other alteration types, such as copy number changes or gene fusions, leaving room for further development. Additional insight into the functional consequences of unknown alterations can be derived from the RNA-seq data, which provide the normalized expression level of both the affected gene and a variant of interest.

### Copy number alterations as diagnostic markers and actionable targets

**Fig. 4:**
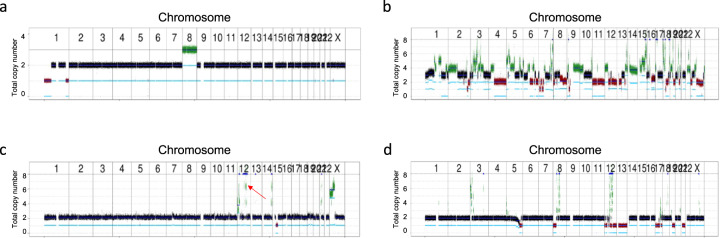
DNA copy number profiles as diagnostic biomarkers. **a** Few DNA copy number changes in a patient with *SS18::SSX2*-positive synovial sarcoma. Consistent with the “silent” genomes of many fusion-driven cancers, a low tumor mutational burden was found with 23 SNVs and indels, corresponding to 0.6 non-synonymous mutations per coding megabase. **b** Multiple DNA copy number alterations in a patient with genomically unstable leiomyosarcoma. **c** Pathognomonic amplification of *MDM2* and *CDK4* on chromosome 12q14-q15 (red arrow), which are targeted by small-molecule inhibitors^68,69^, and few other genomic imbalances in a patient with well-differentiated liposarcoma. **d** Higher genomic complexity with multiple DNA copy number alterations in a patient with dedifferentiated liposarcoma (DDLS). Take-home messages: (i) The extent of DNA copy number alterations varies considerably among tumor types. (ii)The patterns of genomic imbalances can aid in the diagnostic categorization of various cancer entities. (iii) Recurrent amplicons may harbor genes that can be targeted therapeutically, such as *CDK4* and *MDM2* in DDLS.

**Fig. 5:**
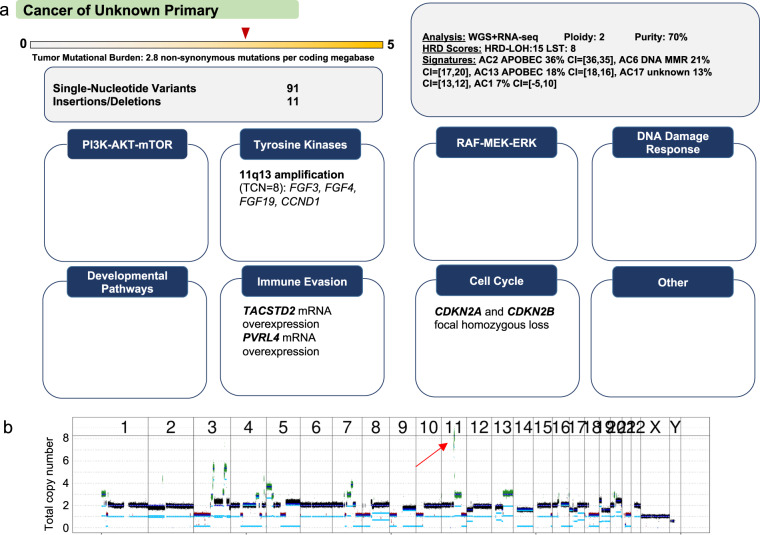
DNA copy number gains as actionable biomarkers. **a** Squamous cell carcinoma of an unknown primary site in the neck region studied by WGS and RNA-seq. MMR, mismatch repair. TCN, total copy number. **b** Evidence of numerous DNA copy number alterations, including amplification (total copy number, 8; average tumor ploidy, 2) of a region on chromosome 11q13.3 containing the oncogenes *CCND1*, *FGF3*, and *FGF4* (red arrow). A query of the OnkoKB precision oncology knowledgebase (www.onkokb.org) showed that data on the oncogenicity of *FGF3* and *FGF4* amplification are inconclusive. RNA-seq analysis showed decreased transcription of *FGF3* and *FGF4*, indicating that their amplification is a passenger alteration. In contrast, *CCND1* was expressed, suggesting that it functions as a driver. The finding of homozygous deletion of *CDKN2A/B* on chromosome 9p21.3 further supported the role of the CCND1–CDK4/6 axis in the pathogenesis of this tumor. However, the clinical efficacy of CDK4/6 inhibition in this scenario varies and appears to be dependent on histology^70,71^. Based on two clinical trials of the CDK4/6 inhibitor palbociclib in combination with cetuximab in *CDKN2A*-negative squamous cell carcinoma of the head and neck region^72,73^, the MTB recommended this treatment with an NCT evidence level of m2a. Chromosomes 1 to Y are indicated. Take-home messages: (i) An amplicon can harbor dozens to thousands of genes that can act as drivers or passengers in a given histologic context. (ii) Integrating WGS/WES and RNA-seq data can guide the selection of driver genes and inform treatment.

**Fig. 6:**
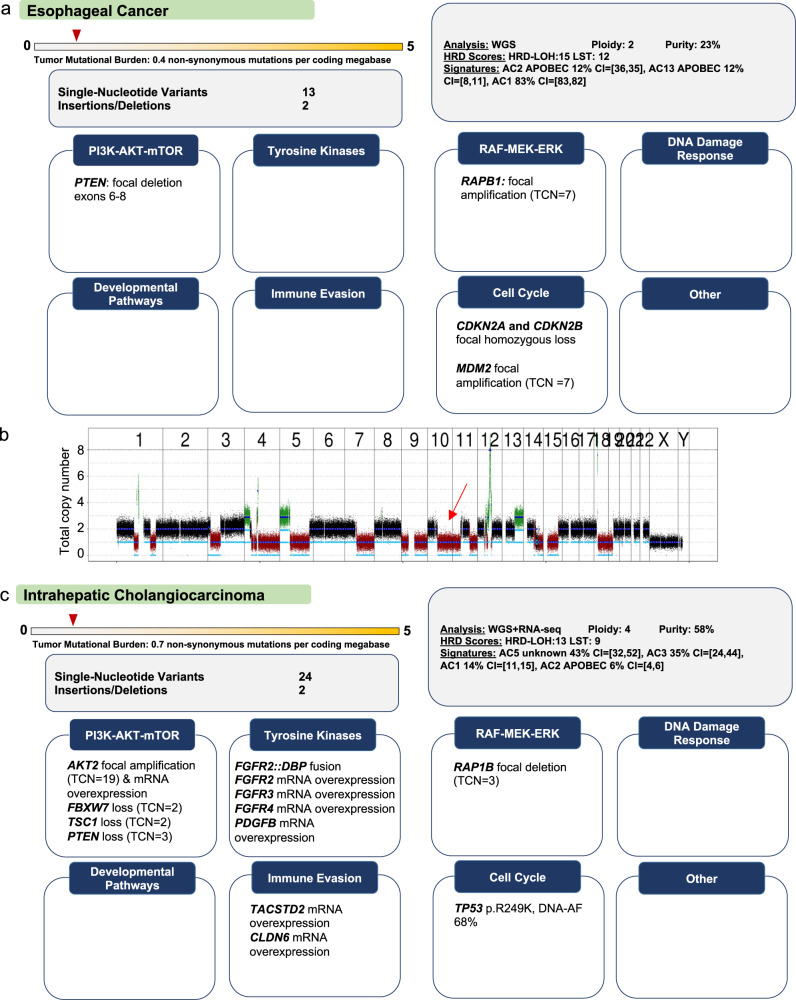
Copy number loss as actionable target. **a** Advanced esophageal cancer studied by WGS and RNA-seq. **b** DNA copy number plot showing a heterozygous deletion of chromosome 10q associated with loss of one *PTEN* allele, which, together with a focal deletion of exons 6 to 8 of the other allele, is predicted to result in loss of PTEN function, providing a rationale for therapeutic inhibition of constitutively active PI3K–AKT–mTOR signaling. Chromosomes 1 to Y are indicated. **c** Intrahepatic cholagiocarcinoma studied by WGS and RNA-seq. In addition to a *FGFR2::DBP* fusion, potentially actionable DNA copy number alterations affecting tumor suppressor genes within the PI3K–AKT–mTOR pathway (average tumor ploidy, 4; *PTEN* copy number, 3; *TSC1* copy number, 2; *FBXW7* copy number, 2) were detected; however, none of these loci was affected by alterations of the remaining allele that would result in complete inactivation, leaving the functional consequences of the copy number losses unclear. RNA-seq showed that all genes were expressed, which, without information on protein expression, argued against treatment recommendations based, at best, on partial inactivation of negative regulators of PI3K–AKT–mTOR signaling. Future integration of proteomic profiling and RNA-based pathway activity estimation into our workflow will provide a more accurate assessment of the impact of DNA copy number alterations on gene function, i.e., their influence on protein synthesis in a given tumor environment. Take-home messages: (i) Heterozygous deletions of tumor suppressor genes without a second “hit” affecting the remaining allele should be interpreted with caution and require further validation, e.g., by immunohistochemistry. (ii) In the case of a deletion affecting one copy of a tumor suppressor gene, examination of the remaining allele, RNA expression, and the integrity of other genes relevant to the respective pathway support the evaluation of functional impact and, thus, clinical actionability.

Since information on genomic gains and losses can guide clinical decision-making, we provide a copy number plot for each patient. For example, the degree and pattern of copy number changes may support the diagnosis of a particular entity. Figure 4 shows that synovial sarcoma, a fusion-driven, genomically “silent” sarcoma, and leiomyosarcoma, characterized by genomic “chaos”, display very different copy number patterns. Furthermore, copy number information can be used to infer the average ploidy of a tumor genome, whose knowledge is essential for the functional and, ultimately, clinical interpretation of genomic imbalances. For example, a focal amplification with a copy number of 6 is less likely to be a tumor-driving alteration if the average ploidy is 4 instead of 2. While global copy number changes, whose patterns were recently categorized into multiple signatures reflecting distinct mutational processes^29,30^, have thus far been primarily of diagnostic value, focal genomic losses, and amplifications can be therapeutic targets. A particular challenge associated with WGS/WES data is the detection of copy number alterations that are focal but still contain tens to hundreds of genes. The delineation of driver and, thus, potentially actionable genes within an amplicon is greatly aided by the whitelisting mentioned above and by integration with RNA-seq data to pinpoint loci whose copy number change leads to altered expression (Fig. 5). In contrast to focal amplifications affecting established oncogenes, the actionability of copy number losses affecting tumor suppressor genes is more difficult to determine, especially when only one gene copy is deleted, and the other allele remains intact (Fig. 6). In the future, such uncertainties may be resolved by integrating pathway analyses inferred from RNA-seq and proteomic data.

### Gene fusions as actionable targets

**Fig. 7:**
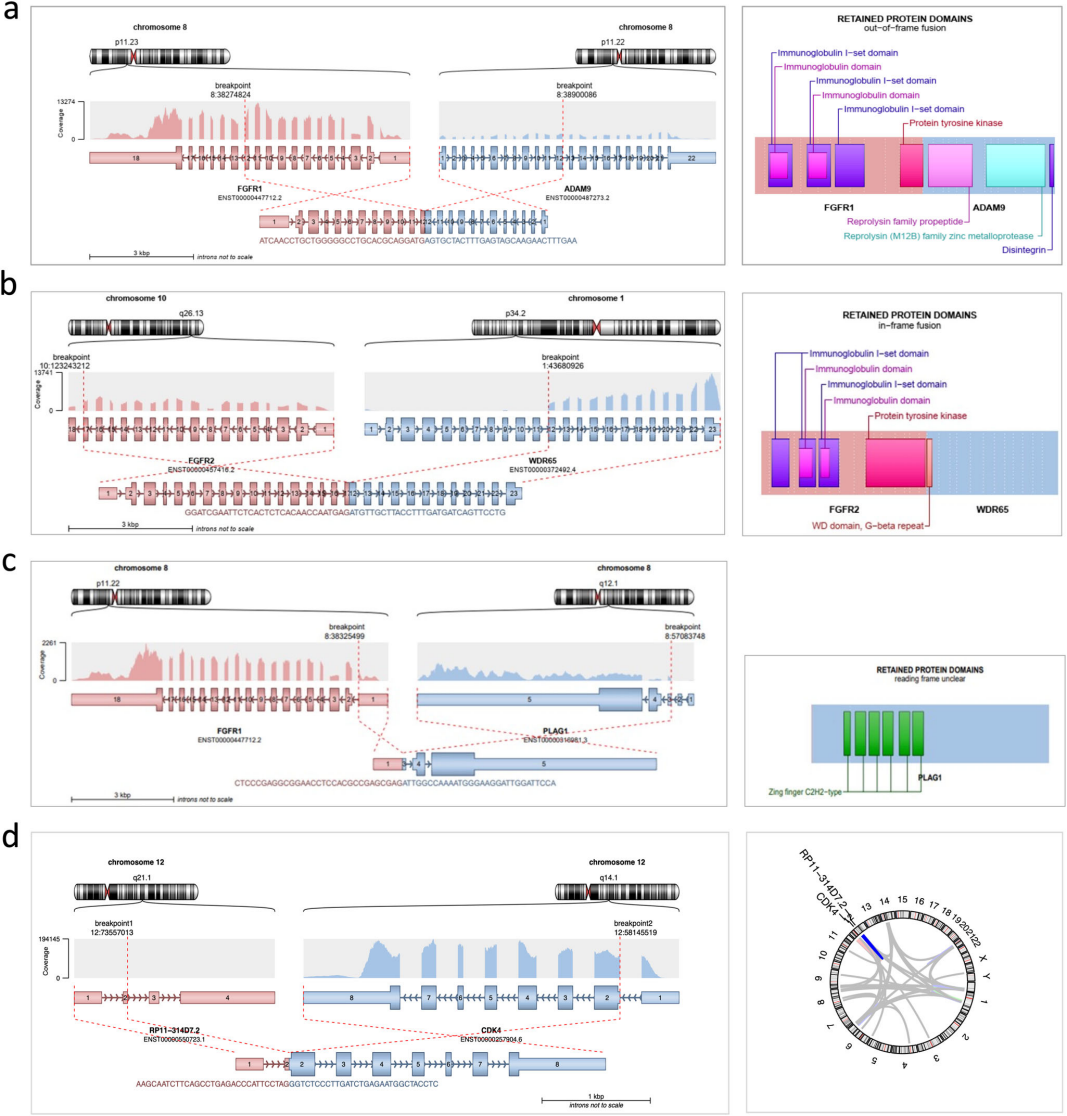
Clinical interpretation of gene fusions. **a** *FGFR1::ADAM9* fusion generated by an interstitial deletion on chromosome 8p linking *FGFR1* exons 1–12 to *ADAM9* exons 12−1 in a patient with chondroblastic osteosarcoma of the femur. As this fusion was out of frame, retained only part of the FGFR1 kinase domain, and was supported by only a few reads, the MTB classified it as non-functional and without therapeutic implications. **b** *FGFR2::WDR65* fusion linking *FGFR2* exons 1−17 to *WDR65* exons 12−23 in a patient with intrahepatic cholangiocarcinoma (iCCC). The chimeric transcript, which is supported by multiple reads for both partners, is characterized by a recurrent breakpoint in *FGFR2* exon 17 and retains the FGFR2 kinase domain. *FGFR2* fusions are identified in approximately 15% of iCCC^74^ and targeted by the recently approved FGFR inhibitor pemigatinib. **c** Oncogenic *FGFR1::PLAG1* fusion in a patient with myoepithelial carcinoma. The genomic breakpoints are located between the promoter and the transcription start site of both *FGFR1* and *PLAG1*, resulting in the expression of full-length *PLAG1* regulated by the *FGFR1* promoter. Accordingly, *PLAG1* but not *FGFR1* was highly expressed, as indicated by the different number of reads. *PLAG1* fusions are characteristic of myoepithelial carcinoma, an aggressive form of salivary gland cancer^75^, and are not yet amenable to targeted therapies. **d** Detection of 184 gene fusions in a patient with DDLS, originating primarily from the alteration of chromosome 12q characteristic of this entity. Gene fusions may be a source of immunogenic neoantigens that can mediate a response to immunotherapy even in tumors with low mutational burden^76^. Take-home messages: (i) RNA-seq is the most accurate method to detect functional gene fusions. (ii) The oncogenicity of a gene fusion does not automatically render the fusion a druggable target. (iii) To date, druggable fusions are mainly restricted to chimeric proteins containing a kinase domain that is constitutively active and triggers downstream signaling.

A major advantage of combined WGS/WES and RNA-seq analysis is the identification of targetable gene fusions that may evade detection by targeted sequencing due to their complexity or breakpoint location, e.g., *NRG1* fusions in KRAS-wildtype pancreatic cancer^31^. For the detection of gene fusions from RNA-seq data, we have developed the Arriba pipeline, which has become a gold standard in terms of accuracy and speed^32–34^. Combined WGS/WES and RNA-seq also allow us to accurately determine the molecular anatomy of gene fusions. This is relevant from a therapeutic perspective since most actionable fusions involve genes encoding kinases, and constitutive kinase activation and “druggability” can be assumed if an open reading frame is created that includes the intact catalytic domain (Fig. 7). Another advantage of including RNA-seq is that one can verify the expression of a fusion gene in the tumor. This information is particularly relevant when evaluating previously undescribed fusions in which an established drug target is joined to a novel partner gene.

### Clinical interpretation of transcriptomic data

**Fig. 8:**
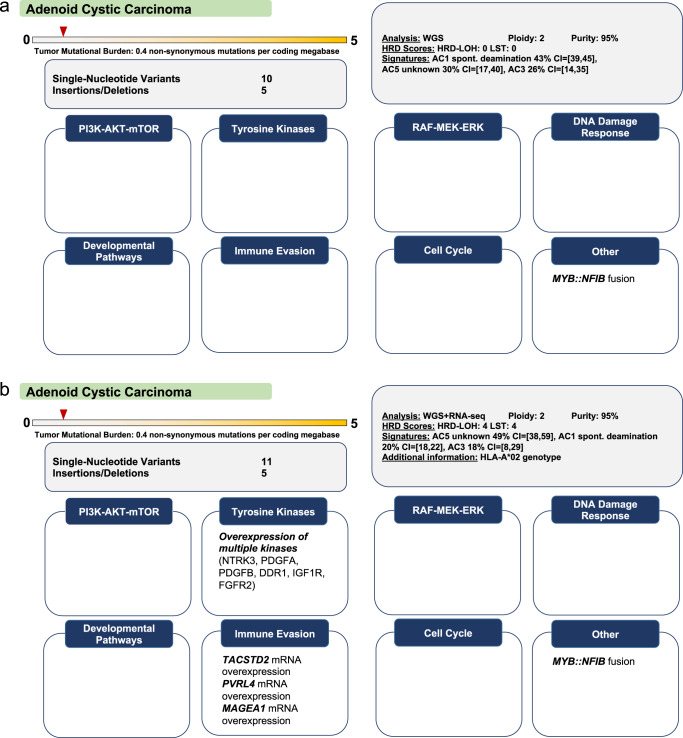
Implications of transcriptome data for guiding cancer therapies. Rationale: These two cases from the same entity highlight the therapeutic value derived from integrating transcriptomic analysis for the emerging list of antibody-drug conjugates and cellular immunotherapy strategies. **a** Adenoid cystic carcinoma (ACC) studied by WGS. The only treatment recommendation was PARP inhibition based on a dominant SBS3 mutational signature. **b** ACC studied by WGS and RNA-seq. In contrast to the previous case, transcriptomic information yielded several treatment recommendations, i.e., sacituzumab govitecan based on overexpression of *TACSTD2*; multi-tyrosine kinase inhibition based on overexpression of *DDR1*, *FGFR2*, *IGF1R*, *PDGFA*, *PDGFB*, and *NTRK3*; T-cell based immunotherapy within a phase 1 clinical trial (ClinicalTrials.gov Identifier: NCT03441100) based on *MAGEA1* overexpression and a HLA-A*02 genotype; and enfortumab vedotin based on *PVRL4* (also called *NECTIN4*) overexpression. Take-home messages: (i) RNA-seq enables the detection of targets for antibody-drug conjugates or cellular immunotherapies. (ii) The selection of kinase inhibitors can be guided through the assessment of their target landscape by RNA-seq.

As described above, analysis of RNA-seq data improves the biological annotation of genetic alterations. In addition, transcriptomic information alone can also yield therapeutic recommendations. First, aberrant expression of kinase genes can guide the use of corresponding inhibitors^35–37^, as exemplified by the identification of candidates for rogaratinib treatment based on *FGFR1-3* expression^38^. Second, gene expression data enable personalized immunotherapy approaches. For example, we frequently identify overexpression of *CLDN6* or *MAGEA4/8*, which prompts eligibility evaluation for appropriate biomarker-stratified clinical trials (ClinicalTrials.gov Identifiers: NCT04503278, NCT03247309). A remaining challenge is the definition of tumor-specific gene expression. We usually report the rank of a gene’s transcript per million value within the MASTER cohort, which, however, can be strongly influenced by a tumor’s location (e.g., primary tumor vs. lung or liver metastasis) and the composition of its microenvironment. Overall, we find that the availability of a transcriptomic data layer significantly increases the number of biologically guided therapy recommendations (Fig. 8).

### Assessment and reporting of germline variants

A major advantage of parallel WGS/WES of tumor and control samples is the ability to directly detect pathogenic germline variants^39^, which are found in approximately 10–15% of cases in the MASTER program and not known before study enrollment in the majority of cases^6,40^. The control sample is usually derived from blood. However, other tissues, e.g., skin, must be used in patients with hematologic neoplasms or after allogeneic stem cell transplantation. The calling of germline variants in cancer predisposition genes, including SNVs, indels, and structural variants, is performed using an open source bioinformatics pipeline at DKFZ^6,24^, and interpretation of filtered rare variants is performed according to the American College of Medical Genetics and Genomics (ACMG) and Association for Molecular Pathology guidelines^41^ and further specifications^42^ by a team of clinical geneticists. For clinical interpretation of germline variants, molecular and clinical characteristics, such as histopathologic diagnosis, age of onset, previous cancers, other phenotypic abnormalities, and especially family history, are considered (Box 1). As this extensive information is not always available during the primary clinical workup, a framework for additional genetic data collection is established. In addition, open questions can be discussed with the treating physician as part of the MTB. The MTB also decides whether a germline finding triggers a recommendation for genetic counseling and must consider the patient’s consent options. If a pathogenic germline variant was detected, a board-certified clinical geneticist or other certified physician with appropriate training should inform the patient about the results and offer formal genetic counseling^43^. Apart from recommendations for genetic testing, pathogenic germline variants can support treatment decisions, e.g., the administration of a PARP inhibitor in germline *BRCA1/2*-mutated pancreatic cancer^44^. An important goal is to harmonize germline variant evaluation across PO programs and improve the data collection and follow-up for patients with genetic tumor risk syndromes.

Box 1: Detection of clinically relevant mosaicism A male patient diagnosed with leiomyosarcoma of the mesenterial fat at age 59 years was enrolled in the MASTER program due to progressive disease on doxorubicin and olaratumab and on gemcitabine and docetaxel. WGS revealed 30 SNVs and one indel, including variants in *TP53* (p.H193R; AF, 0.79) and *RB1* (p.F839fs*10; AF, 0.81), which were both associated with loss of heterozygosity of the wildtype allele, a typical finding in leiomyosarcomas that show near-universal inactivation of *TP53* and *RB1*^77^. Illustrating the value of paired tumor and matched normal tissue analysis, the *RB1* indel was detected in the control sample with an allele frequency of 5.7%. The medical history revealed that enucleation was performed at the age of three years due to an eye tumor, and genetic counseling was recommended due to the very likely presence of a pathogenic *RB1* variant as mosaicism. *RB1* mosaicism occurs in approximately 5% of parents of children with unilateral retinoblastoma^78^. The degree of mosaicism in different tissues is difficult to assess, but the variant may be inherited up to 50%. This should be considered during treatment, especially irradiation, due to an increased risk of secondary malignancies (38% vs. 21% by age 50 in irradiated vs. non-irradiated patients^79^).

### Assignment of evidence to actionable biomarkers and treatment recommendations

**Fig. 9:**
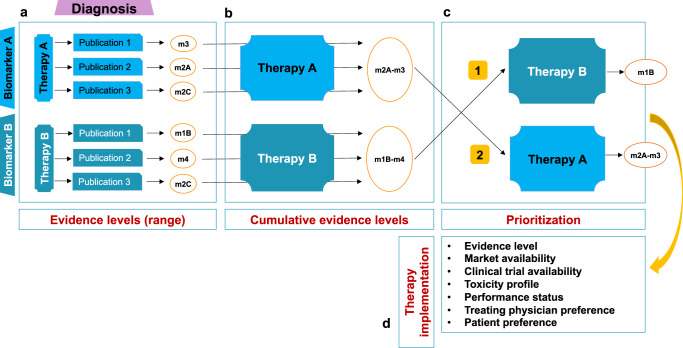
Assignment and ranking of biomarker-drug response associations. The number of molecular alterations identified by multiomics is steadily increasing, which entails two main challenges, i.e., determining the biological significance and clinical relevance of a candidate biomarker. **a** The evidence supporting a molecularly informed therapy ranges from preclinical studies to phase 3 clinical trials or meta-analyses. The underlying histologic entity must always be considered when assigning evidence to a molecular biomarker. In the case presented, therapy A is supported by clinical data from other entities (m2a), whereas for therapy B, there are retrospective data from the same entity supporting the recommendation (m1b). **b** The final evidence attributed to treatment can be listed as a range between the lowest and highest level, (e.g., m2a–m3). The number of references listed varies among curators, but we advise careful judgment and restriction to the most contextually relevant ones. **c**, **d** Prioritization of therapies is a complex process and depends on several variables that must be considered in a patient’s specific clinical and socioeconomic situation at a given time. For example, a therapy may be available in a clinical trial, but exclusion criteria beyond molecular features preclude enrollment, and another treatment option must be pursued.

We recently described our approach to assigning evidence to biomarker-drug response associations^14^ and the variant classification system developed at NCT, which is used by the major precision oncology networks in Germany^15^. There are four NCT evidence levels: m1, evidence in the same entity; m2: evidence in a different entity; m3, preclinical evidence; m4, biological rationale. Levels m1 and m2 have three suffixes denoting the study type from which the evidence was derived: A, prospective study or meta-analysis; B, retrospective cohort or case-control study; C, case study or single unusual responder. Treatment recommendations are drafted before the MTB and are primarily based on evidence for associations between molecular biomarkers and drug response, taking into account tumor entity (Fig. 9). We do not limit our recommendations to approved drugs, as compounds in clinical development may become available in the short to medium term. Alternatively, a patient’s molecular findings would need to be regularly reevaluated in an MTB, which is currently not feasible due to the increasing number of cases and limited automation of clinical decision-making to date. All recommendations are based on both knowledgebase entries and an extensive manual literature search and always include suitable biomarker-stratified trials if available.

### Molecular tumor board discussion

Given the increasing number of patients enrolled in the MASTER trial and the related CATCH (Comprehensive Assessment of Clinical Features and Biomarkers to Identify Patients with Advanced or Metastatic Breast Cancer for Marker-Driven Trials in Humans) program for metastatic breast cancer^45^, two MTBs are held at NCT Heidelberg each week that focus on clinical decision-making based on WGS/WES, RNA-seq, and methylome data and last, on average, two hours. Participants include treating physicians, molecular oncologists, pathologists, clinical geneticists, and clinical bioinformaticians. The MTBs are multicentric, including, e.g., participants from all partner sites of the German Cancer Cancer Consortium. Each case discussion begins with a presentation of the clinical history by the treating physician, followed by a summary of the molecular alterations by a clinical bioinformatician. Next, the molecular oncologist responsible for the clinical interpretation of the multiomics profile presents and assigns a level of evidence to the resulting recommendations and concludes with a proposed ranking of treatment options. Finally, clinical geneticists evaluate and classify the germline variants detected, supported by the personal and family histories provided by the treating clinician, followed by a recommendation for genetic counseling if indicated. Similar to conventional, entity-specific tumor boards, MTBs regularly discuss matters pertaining to a patient’s performance status and previously administered therapies. In this context, the ranking of molecularly informed therapy options is particularly important and consequently accounts for a relevant part of the discussion. When available, molecular biomarker-stratified clinical trials are generally prioritized over off-label therapies. However, there are examples where the latter are ranked higher, either when the evidence level is higher or when other criteria, e.g., clinical performance status, prevent the patient from being enrolled in a trial. Due to its multicenter structure, the MTB also provides an ideal forum for the regular dissemination of information about new trials across Germany. Every case presentation, which typically lasts eight to ten minutes, ends with a consensus on the recommended treatment options.

### Molecular tumor board report

**Fig. 10:**
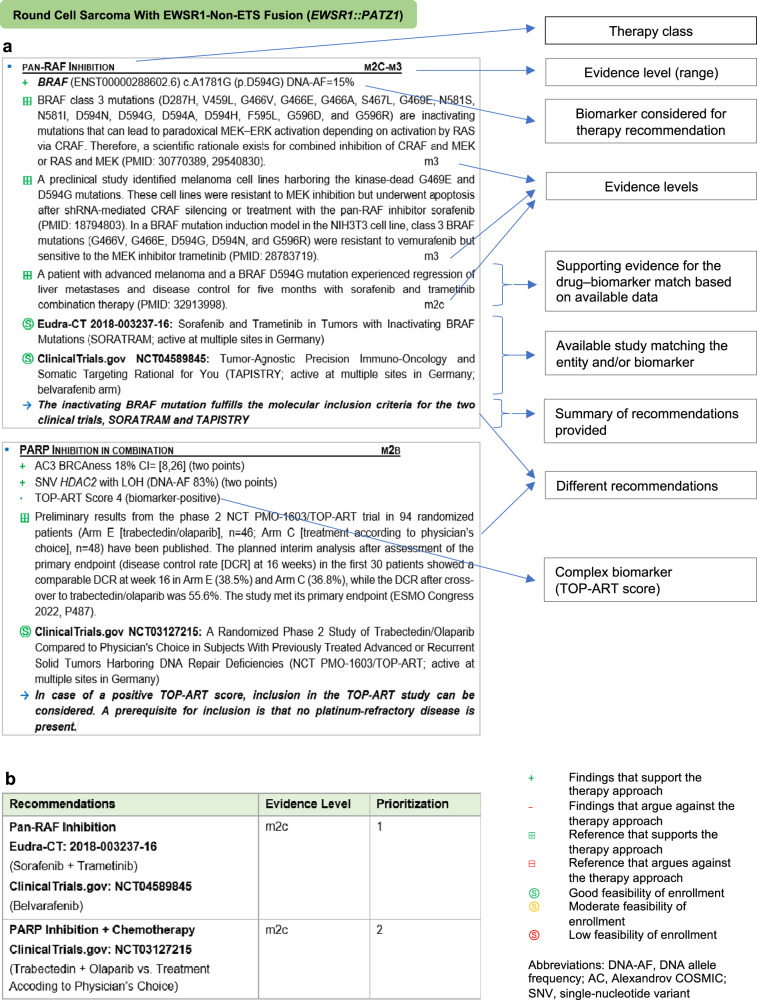
Structure of an MTB report. **a** Main components of a therapy recommendation block as used in the MASTER MTB report. **b** Summary and ranking of therapy recommendations with their respective evidence levels. Several factors influence the final choice of therapy, such as its availability, patient preference, side effects, and approval status.

The MTB report, which summarizes treatment recommendations based on the multiomics data, begins with a summary of the disease course and previous therapy. Over the years, we have developed a structure for reporting treatment recommendations that has proven to be a comprehensive basis for clinical decision-making (Fig. 10). Recommendations are organized into blocks, each reflecting a specific therapy approach. They begin with a list of detected biomarkers of response or resistance to the respective treatment, followed by evidence supporting the particular entity-biomarker-drug association. Where available, biomarker-stratified clinical trials are provided. Each block concludes with a summary and synthesis of the evidence for and against a therapeutic strategy. At the end of the report, a table summarizes all recommendations and the prioritization decided on in the MTB. Of the germline variants, only those assigned to ACMG classes 4 and 5 are included. Finally, the consented MTB report is sent to the treating oncologist, who takes further steps regarding patient counseling and therapy implementation.

## Discussion

The MASTER trial continues to evolve regarding both the dimensions in which individual tumors are studied and the bioinformatics workflow linked to multidimensional profiling. Emerging diagnostic layers include (phospho)proteomics^46^, drug sensitivity profiling in primary cell lines and organoids, tumor microenvironment analyses, digital pathology, radiomics, and liquid biopsies. The bioinformatics pipeline has recently been extended to include, e.g., elements that allow the prediction of pharmacogenomic risk and immunogenic neoepitopes, as well as tumor telomere status^47^. To develop additional predictive biomarkers based on integrative data analyses, we are increasingly pursuing systems biology approaches, focusing on the functional taxonomy of tumors and signaling pathway activities^48,49^ that might be exploited therapeutically. Furthermore, we aim to leverage the potential of WGS by exploring alterations in intergenic regions that may have clinical implications.

Due to the inclusion criteria of the MASTER program, i.e., advanced cancers in young adults and rare malignancies, we currently use multiomics profiling in only a fraction of all cancer patients. A critical issue on the way to comprehensive profiling in all cancer patients is the scalability of the diagnostic workflow through automation, especially of high-level operations that follow the primary acquisition and bioinformatic processing of raw data. To this end, we are developing the Knowledge Connector, a customized software suite that supports the four major components of the MTB workflow, i.e., (i) preparation, including the linkage of a patient’s clinical and molecular data with information from external databases and the growing collection of in-house cases and the documentation and evidence grading of treatment recommendations; (ii) presentation of relevant clinical and molecular data, their association, and the resulting recommendations, including supporting evidence; (iii) semi-automated issuing of an MTB report; and (iv) MTB organization, including patient enrollment, participant documentation, and direct links to individual case presentations.

Another issue is that access to molecularly guided off-label therapies may be more likely in rare cancers than in common entities for which more evidence-based standard treatments exist. Notwithstanding this consideration, the potential of multiomics-guided PO to improve patient outcomes is best realized by systematically testing the clinical value of new biomarkers. Hence, a major effort is underway at NCT to develop a portfolio of molecularly stratified clinical trials as part of the NCT Precision Medicine in Oncology (PMO) Program, which currently includes the NCT PMO-1601 (ClinicalTrials.gov Identifier: NCT03110744)^50^, NCT PMO-1602/CRAFT (NCT04551521)^51^, NCT PMO-1603/TOP-ART (NCT03127215)^52^, and NCT PMO-1604 (NCT04410653) protocols.

## Methods

### Multilayered tumor profiling in the MASTER trial

**Fig. 11:**
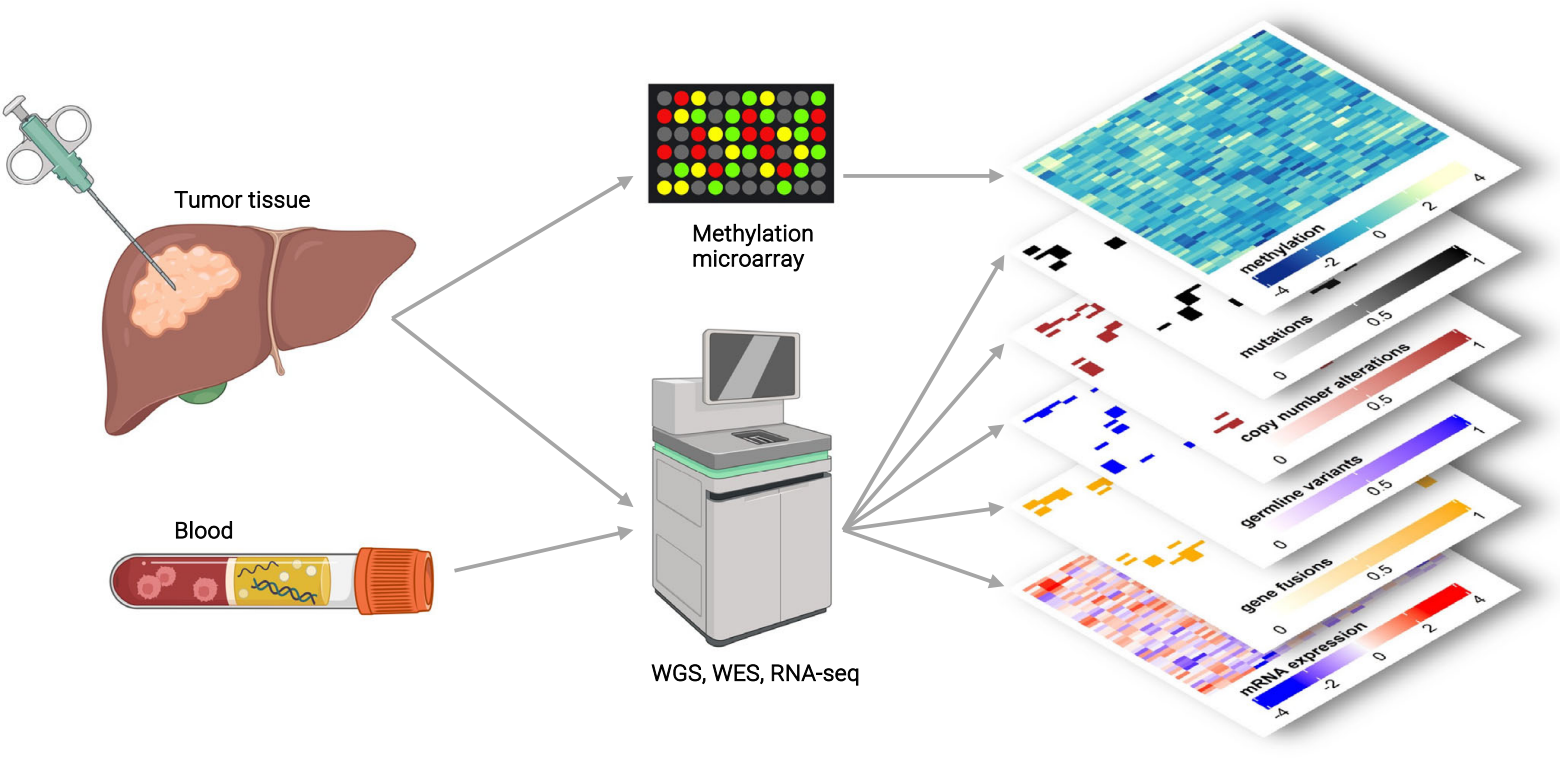
Graphical representation of multidimensional tumor characterization as performed in the MASTER trial. Tumor DNA and RNA obtained from tumor tissue are analyzed by DNA methylation profiling and WGS/WES or RNA-seq, respectively. DNA derived from blood serves as a matched normal control for WGS/WES. Created with Biorender.com.

MASTER is a prospective, continuously recruiting, multicenter observational study for biology-guided stratification of adults with rare cancers, including rare subtypes of common entities, using comprehensive molecular profiling, and clinical decision-making in a multidisciplinary MTB^53^. The study is conducted in accordance with the Declaration of Helsinki and the protocol (S-206/2011) was approved by the Ethics Committee of the Medical Faculty of Heidelberg University. The diagnostic workflow (Fig. 11) starts with patient registration and obtaining informed consent for sample acquisition and molecular analysis, including tiered consent for germline analysis. Tumor tissue is obtained through resection or biopsy, and a minimum tumor cell content of 20%, evaluated by a pathologist, is required for further analysis. In parallel, a blood sample is collected to enable comparative analysis of the germline genome. Processing of tissue and blood specimens, as well as WGS/WES, RNA-seq, and array-based DNA methylation profiling, are performed under accredited conditions in a dedicated NCT/DKFZ Sample Processing Laboratory and the DKFZ Genomics and Proteomics Core Facility, respectively. Here, minimum coverage in the tumor (WGS, 80x; WES, 120x; RNA-seq, 30 million reads) and control (WGS, 40x; WES, 80x) samples is ensured. Further technical details were reported recently^6^. Methylome data are generated using Infinium MethylationEPIC BeadChip technology (Illumina, #WG-317) following the manufacturer’s instructions. The raw data obtained for a sample can exceed one terabyte and are first processed through an automated bioinformatics workflow established at DKFZ^54^, followed by annotation of molecular alterations by clinical bioinformaticians at NCT and DKFZ using in-house pipelines and various knowledge bases and tools (Table 1).

**Table 1:** Knowledge bases, software tools, and guidelines used in clinical interpretation of multiomics data.

Resource	Reference	Application
Cancer Hotspots	^55^	Detection of recurrent single-nucleotide variants (SNVs) and small insertions and deletions (indels)
ClinVar	^56^	Annotation of germline variants
cBioPortal for Cancer Genomics	^57,58^	Detection of recurrent SNVs and indels; visualization of molecular data
Catalogue Of Somatic Mutations In Cancer (COSMIC)	^59^	Detection of recurrent SNVs and indels
Jackson Laboratory Clinical Knowledgebase (JAX-CKB)	^60^	Biological classification of molecular alterations; treatment recommendation; clinical trial matching
NCT Precision Oncology Thesaurus Drugs	^61^	Translation between drug targets, drugs, and drug classes; assessment of drug-target interactions and pharmacodynamic equivalence; treatment recommendation
VarSome	^62^	Annotation of somatic and germline variants
Variant Interpretation for Cancer Consortium (VICC) standard operating procedure	^28^	Classification of somatic variant oncogenicity

### Reporting summary

Further information on research design is available in the Nature Research Reporting Summary linked to this article.

### Supplementary information

REPORTING SUMMARY

## Data Availability

The data presented in Fig. 4 were generated from processed beta values deposited in the Gene Expression Omnibus public repository under accession number GSE140686.
